# Facts from Correspondents

**Published:** 1846-09

**Authors:** 


### FACTS FROM CORRESPONDENTS.

---

We make the following extracts from a letter for which we are indebted to R. A. Westbrook, M.D., of Oakland, Tennessee.

We have had a healthy spring and summer. The spring was cold and wet, and for a time bowel complaints of a mild character prevailed. At this time scarlatina may be said to be endemic in this region, and sporadic cases have been occurring for many weeks; the cases that have fallen in my way have been mild and easily controlled by remedial treatment, but a number have assumed a malignant type and terminated speedily in death. This, I believe, is characteristic of scarlet fever; in one instance so mild as to require no treatment; in another resisting all remedies, and terminating fatally in a few hours. Cholera is scarcely more rapid in its march than some cases of scarlatina; and again physicians will meet with a long succession of mild cases, and practise a whole season without losing a patient with this fever. Remittent fever made its appearance early in the summer and attained, during a spell of hot, dry weather, considerable prevalence, but we had several drenching rains a fortnight since, by which it has been checked. I do not learn that any new cases have occurred since. During all the season we have had occasional cases of intermittent fever. I remarked in the spring a tendency in the submaxillary glands to become inflamed and swollen from any slight injury of the system, and often without any assignable cause. Some weeks ago a catarrhal fever prevailed among children, and a number of cases proved fatal under the management of steam-doctors. One practitioner of this school lost three patients with this affection in eleven hours. He confined them to heated rooms, steamed them, and gave them lobelia till they died. He remarked to the friends that the "children could take as much lobelia as men." No other cases of this complaint had an unfavorable issue in this neighborhood, and the result in these, I suppose, is attributable to the barbarous practice pursued.

August 4th, 1846.

